# Statistics of Medical Schools—1849–50

**Published:** 1850-07

**Authors:** 


*Statistics of Medical Schools*—1849–50.

| | *Students.* | *Graduates.* |
|---|---|---|
| University of Pennsylvania, - - - - | 438 | |
| University of the City of New-York, - - | | 111 |
| Harvard University, - - - - - | | 23 |
| College of Physicians and Surgeons, N. Y., - - | | 49 |
| Medical College of Ohio, - - - - | | 42 |
| Buffalo University, - - - - - | 115 | 27 |
| Yale College, - - - - - - | | 16 |
| Rush Medical College, - - - - | 120 | 44 |
| Albany Medical College, - - - - | 100 | 26 |
| Uuiversity of Louisville, - - - - | 376 | 113 |
| Transylvania University, - - - - | 92 | 32 |
| University of St. Louis, - - - - | I10 | |
| University of Missouri, (St. Louis), - - - | I10 | |
| Medical College of Evansville, - - - | 40 | 6 |
| Indiana Central Medical College, - - - | 50 | 10 |
| Starling Medical College, - - - - | 154 | |
| Jefferson Medical College, - - - - | 516 | 211 |
| Pennsylvania Medical College, - - - | 106 | 34 |
| Medical College of Georgia, - - - | 179 | 44 |
| Berkshire Medical College, - - - - | 93 | 23 |

—*North Western Med. and Surg. Journal.*

